# A replication study of genetic risk loci for ischemic stroke in a Dutch population: a case-control study

**DOI:** 10.1038/s41598-017-07404-4

**Published:** 2017-09-22

**Authors:** Allard J. Hauer, Sara L. Pulit, Leonard H. van den Berg, Paul I. W. de Bakker, Jan H. Veldink, Ynte M. Ruigrok, Dutch Parelsnoer Institute-Cerebrovascular accident (CVA) Study Group, Catharina J. M. Klijn, Ale Algra, Ewoud J. van Dijk, Peter J. Koudstaal, Gert-Jan Luijckx, Paul J. Nederkoorn, Robert J. van Oostenbrugge, Marieke C. Visser, Marieke J. Wermer, L. Jaap Kappelle

**Affiliations:** 1 Department of Neurology and Neurosurgery, Brain Center Rudolf Magnus, University Medical Center Utrecht, Utrecht, The Netherlands; 2 Department of Genetics, Center for Molecular Medicine, University Medical Center Utrecht, Utrecht, The Netherlands; 3 Department of Epidemiology, Julius Center for Health Sciences and Primary Care, University Medical Center Utrecht, Utrecht, The Netherlands; 4 The Dutch Parelsnoer Institute-Cerebrovascular accident (CVA) Study Group, Utrecht, The Netherlands; 5 Department of Neurology, Donders Institute of Brain Behaviour & Cognition, Center for Neuroscience, Radboud University Medical Center, Nijmegen, The Netherlands; 6 Department of Clinical Epidemiology, Leiden University Medical Center, Leiden, The Netherlands; 7 Department of Neurology, Erasmus Medical Center, Rotterdam, The Netherlands; 8 Department of Neurology, University Medical Center Groningen, Groningen, The Netherlands; 9 Department of Neurology, Academic Medical Center, Amsterdam, The Netherlands; 10 Department of Neurology, Maastricht University Medical Center, Maastricht, The Netherlands; 11 Department of Neurology, VU Medical Center, Amsterdam, The Netherlands; 12 Department of Neurology, Leiden University Medical Center, Leiden, The Netherlands

## Abstract

We aimed to replicate reported associations of 10 SNPs at eight distinct loci with overall ischemic stroke (IS) and its subtypes in an independent cohort of Dutch IS patients. We included 1,375 IS patients enrolled in a prospective multicenter hospital-based cohort in the Netherlands, and 1,533 population-level controls of Dutch descent. We tested these SNPs for association with overall IS and its subtypes (large artery atherosclerosis, small vessel disease and cardioembolic stroke (CE), as classified by TOAST) using an additive multivariable logistic regression model, adjusting for age and sex. We obtained odds ratios (OR) with 95% confidence intervals (95% CI) for the risk allele of each SNP analyzed and exact p-values by permutation. We confirmed the association at 4q25 (*PITX2*) (OR 1.43; 95% CI, 1.13–1.81, p = 0.029) and 16q22 (*ZFHX3*) (OR 1.62; 95% CI, 1.26–2.07, p = 0.001) as risk loci for CE. Locus 16q22 was also associated with overall IS (OR 1.24; 95% CI, 1.08–1.42, p = 0.016). Other loci previously associated with IS and/or its subtypes were not confirmed. In conclusion, we validated two loci (4q25, 16q22) associated with CE. In addition, our study may suggest that the association of locus 16q22 may not be limited to CE, but also includes overall IS.

## Introduction

A substantial proportion of the etiology of acute ischemic stroke (IS) is thought to be attributable to (common) genetic variation^1^. Genome-wide association studies (GWAS) have estimated that the proportion of phenotypic variance of IS explained by common variants ranges between 16 and 40%, depending on subtype^1^. Thus far, GWAS have identified a small number of single nucleotide polymorphisms (SNPs) associated with overall IS or its subtypes large artery atherosclerosis (LAA), small vessel disease (SVD) and cardioembolism (CE)^2–11^. These loci have been suggested to be mostly or entirely subtype specific. Discovery of common variants influencing stroke is hindered by many challenges, including but not limited to the heterogeneity of the phenotype, high lifetime risk of stroke, late disease onset, and limited statistical power in studies performed to date. Thus, replication of presumed risk loci in independent cohorts is emphatically recommended before initiating fine-mapping efforts in search of causal variants and functional studies to discern the functional consequences of these variants^12^. We aimed to replicate the associations of eight loci with IS and/or its subtypes as previously reported in an independent set of patients with IS drawn from a Dutch cohort.

## Methods

### Participants

We included 1,375 patients with IS of Dutch descent who were enrolled in the Dutch Parelsnoer initiative (PSI) Cerebrovascular Disease^13^. This study represents an ongoing collaboration of eight university medical centers in the Netherlands in which clinical data, imaging and biomaterials of patients with stroke are prospectively and uniformly collected^13^. The present study includes patients with IS enrolled between September 2009 and November 2014. IS was defined as focal neurologic deficits of sudden onset originating from the brain and persisting for more than 24 hours, in the absence of hemorrhage as confirmed by imaging. We further classified IS subtypes according to the Trial of Org 10172 in Acute Stroke Treatment (TOAST), LAA, SVD, CE, and stroke of other and of undetermined cause^14^. We used 1,533 population-level controls of Dutch descent^15^. Information on ancestry in patients and controls was obtained by self-report. The Medical Ethics Committee of the University Medical Center Utrecht approved the study and all patients provided written informed consent. The research described was conducted in accordance with relevant guidelines and regulations.

### Genotyping

DNA of the cases and controls was extracted from peripheral blood. We genotyped 10 SNPs of eight loci (1p13.2 (*TSPAN2*), 4q25 (*PITX2*), 6p21.1 (*SUPT3H/CDC5L*), 7p21.1 (*HDAC9*), 9p21.3 (*CDKN2BAS1*), 9q34 (*ABO*), 12q24 (*ALDH2*) and 16q22 (*ZFHX3*)) using KASP assays (LGC Genomics, Hoddesdon, UK).

### Statistical analysis

We removed individuals with >25% missing genotypes (29 individuals; 7 cases and 22 controls). We tested each SNP for deviation from Hardy-Weinberg equilibrium (p < 0.001) and calculated minor allele frequencies for each SNP in cases and controls. As the design of the study prevented us from performing principal component analyses to test for ancestral homogeneity, we compared risk allele frequencies with those from the Genome of the Netherlands (GoNL) Project^16^. GoNL comprises a comprehensive characterization of genetic variation of 769 individuals of Dutch ancestry as assessed by whole-genome sequencing^16^. Frequencies were calculated in the unrelated set of individuals in GoNL (N = 498). Next, we tested these SNPs for association with IS and its subtypes using an additive logistic regression model, which includes 0, 1 or 2 copies of the risk alleles, and adjusted for age and sex. We report odds ratios (OR) with 95% confidence intervals (95% CI) for each risk allele as established in previous studies^2,4^. To assess the validity of also including samples with missing genotypes, we performed a sensitivity analysis excluding each individual with at least one missing genotype. Accompanying exact probability-values for the observed associations were obtained by performing 10,000 permutations. Analyses were performed in Plink version 1.9b3.38.

### Data Availability

The datasets generated during and/or analysed during the current study are available from the corresponding author on reasonable request.

## Results

After quality control, our data set consisted of 1,368 IS patients (803 (58.7%) men, median age 67.3 years (interquartile range (IQR): 56.5–77.2)) and 1,511 controls of Dutch descent (926 (61.3%) men, median age 64.4 years (IQR: 58.0–70.3)). Baseline characteristics of the patients with IS are presented in Supplementary Table S1. All genotyped SNPs had call rates >98% and were in Hardy-Weinberg equilibrium. Risk allele frequency of all variants showed high concordance with those reported in the Genome of the Netherlands Project (Table 1)^16^.

**Table 1:** Results of replication of previously established SNPs with acute ischaemic stroke and its subtypes.

Phenotype	SNP	Nearest gene	Chr.	Base pair position	Previously reported association	Ref.	Cases/Controls	Risk allele	RAF GoNL	RAF cases/controls	Power (%)	Adj. OR (95% CI)^*^	p-value^†^
Overall IS	rs2200733	*PITX2*	4q25	110789013	1.25 (1.15–1.37), p = 3.14 × 10^−7^	^3^	1354/1473	T	0.10	0.11/0.10	34	1.11 (0.94–1.31)	0.90
Overall IS	rs505922	*ABO*	9q34	136149229	1.07 (1.03–1.11), p = 0.0006	^8^	1358/1477	C	0.34	0.34/0.32	47	1.09 (0.97–1.22)	0.74
Overall IS	rs10744777	*ALDH2*	12q24	111795214	1.10 (1.07–1.13) p = 7.12 × 10^−11^	^9^	1359/1462	T	0.65	0.66/0.66	6	1.01 (0.90–1.12)	1.00
Overall IS	rs7193343	*ZFHX3*	16q22	72995261	1.11 (1.04–1.17), p = 0.00054	^10^	1364/1487	T	0.17	0.20/0.17	98	1.24 (1.08–1.42)	0.016
LAA	rs12122341	*TSPAN2*	1p13.2	115113069	1.19 (1.12–1.26), p = 1.30 × 10^–9^	^2^	360/1479	G	0.26	0.26/0.24	20	1.09 (0.90–1.32)	0.99
LAA	rs556621	*SUPT3H/CDC5L*	6p21.1	44626422	1.21 (1.13–1.30), p = 4.7 × 10^−8^	^5^	360/1484	T	0.30	0.28/0.32	29	0.89 (0.44–1.06)	0.86
LAA	rs11984041	*HDAC9*	7p21.1	18992312	1.42 (1.28–1.57), p = 1.87 × 10^−11^	^4^	362/1477	T	0.09	0.13/0.10	81	1.33 (1.04–1.72)	0.22
LAA	rs2107595	*HDAC9*	7p21.1	19009765	1.39 (1.27–1.53), p = 2.03 × 10^−16^	^11^	359/1476	A	0.14	0.20/0.17	72	1.22 (0.99–1.51)	0.43
LAA	rs2383207	*CDKN2B-AS1*	9p21.3	22115960	1.16 (1.04–1.29), p = 0.0083	^7^	362/1486	G	0.50	0.54/0.49	82	1.24 (1.05–1.46)	0.11
LAA	rs505922	*ABO*	9q34	136149229	1.23 (1.07–1.18), p = 0.001	^8^	359/1477	C	0.34	0.35/0.32	89	1.15 (0.97–1.37)	0.65
SVD	rs10744777	*ALDH2*	12q24	111795214	1.17 (1.11–1.23), p* = *2.92 × 10^−9^	^2^	285/1462	T	0.65	0.68/0.66	16	1.08 (0.88–1.30)	1.00
CE	rs2634074	*PITX2*	4q25	110755885	1.33 (1.24–1.42), p = 1.52 × 10^−16^	^9^	220/1482	T	0.18	0.26/0.19	95	1.43 (1.13–1.81)	0.0285
CE	rs2200733	*PITX2*	4q25	110789013	1.54 (1.33–1.78), p = 8.05 × 10^−9^	^3^	220/1473	T	0.10	0.13/0.10	56	1.30 (0.96–1.76)	0.60
CE	rs505922	*ABO*	9q34	136149229	1.13 (1.11–1.15), p = < 0.001	^8^	219/1477	C	0.34	0.35/0.32	52	1.20 (0.96–1.49)	0.65
CE	rs7193343	*ZFHX3*	16q22	72995261	1.22 (1.10–1.35), p* = *0.00021	^10^	221/1487	T	0.17	0.24/0.17	100	1.62 (1.26–2.07)	0.0014

SNP; single nucleotide polymorphism; Chr, chromosome; LAA, large artery atherosclerosis; IS, ischemic stroke; CE, Cardioembolism; SVD, small vessel disease; Ref, reference; RAF, risk allele frequency; GoNL, Genome of the Netherlands; Adj, adjusted; OR, odds ratio; CI, confidence interval.

*Adjusted for sex and age.

^†^Calculated by permutation.

At the 4q25 (*PITX2)* locus, previously identified by GWAS, we confirmed the association at rs2634074 with CE stroke (OR = 1.43 for the T allele; 95% CI, 1.13–1.81, p = 0.029), but not at rs2200733 (OR = 1.30 for the T allele; 95% CI, 0.96–1.76; p* = *0.60) (Table 1). We also replicated 16q22 (*ZFHX3*) as a risk locus for CE (OR = 1.62 for the T allele; 95% CI, 1.26–2.07, p = 0.0014) and found this locus significantly associated with overall IS (OR = 1.28 for the T allele; 95% CI, 1.12–1.47, p = 0.002) (Table 1). This association remained significant in a sensitivity analysis excluding cases with cardioembolic stroke (OR = 1.18; 95% CI, 1.03–1.36, p = 0.02), and when we only included patients with LAA stroke or SVD (OR = 1.22; 95% CI, 1.03–1.45, p = 0.02).

We could not replicate the previously-established associations at the 1p13.2 (*TSPAN2*), 6p21.1 (*SUPT3H/CDC5L*), 7p21.1 (*HDAC9*), 9p21.3 (*CDKN2B-AS1*), 9q34 (*ABO*) and 12q24 (*ALDH2*) loci with overall IS or its subtypes. However, all of the effect directions were consistent with the observed directions of the initial association reports except for the C allele of rs556621 at locus 6p21.1 (*SUPT3H/CDC5L*) (Table 1).

Results did essentially not differ when individuals with missing genotypes were excluded (Supplementary Table S2).

## Discussion

In a well-defined cohort of patients with IS, we confirmed the 4q25 (*PITX2*) and 16q22 (*ZFHX3*) loci to be significantly associated with the IS subtype CE. We also found locus 16q22 to be significantly associated with overall IS. We were not able to replicate the previously-established associations at the 1p13.2 (*TSPAN2*), 6p21.1 (*SUPT3H/CDC5L*), 7p21.1 (*HDAC9*), 9p21.3 (*CDKN2B-AS1*), 9q34 (*ABO*) and 12q24 (*ALDH2*) with overall IS or its subtypes although, barring locus 6p21.1 (*SUPT3H/CDC5L*), the effect direction of their associations were consistent with expectation^2–11^.

Previous studies have consistently demonstrated the association between variants at 4q25 (*PITX2*) and 16q22 (*ZFHX3*) with atrial fibrillation both in patients with and without IS^3,10^, and additionally with cardioembolic stroke^2–4^. In the 4q25 locus, we only replicated rs2634074, but not rs2200733, despite moderate linkage disequilibrium (r^2^ = 0.51) and a comparable effect size as established previously^2–4,9^. This finding is likely explained by the difference in the power to detect a statistically-significant signal at each SNP (95% and 56%, respectively), a difference that results from their ~10% frequency difference. After the initial report of the association^10^, other studies found locus 16q22 to be specific for CE^2,4^, whereas our findings point to a possible association with both CE and overall IS. The association with overall IS remained significant after excluding cases with cardioembolic stroke, possibly suggesting a partially shared genetic architecture across different stroke subtypes^17^.

Variants near *PITX2* that encode for a transcription factor have convincingly been implicated in sinoatrial node development and regulation of cardiac action potentials^18^. Little is known about the role of *ZFHX3* in ischemic stroke. Besides atrial fibrillation, this gene has also been implicated in the regulation of myogenic and neuronal differentiation, and as a tumor suppressor gene in multiple cancers. Additionally, sequence variants in the locus have been linked to Kawasaki disease^10^. These lines of evidence may suggest that the role of this locus in IS might not be restricted to those of cardiac origin, and therefore may explain its potential association with overall IS.

While recent publications have suggested that implicated variants are likely subtype specific^2,4^, it is noteworthy that some genetic overlap between diagnostic IS subtypes has also been reported^17^. Given the repeated discovery of this locus in cardioembolic stroke in large-scale GWAS, it is likely that the observed association of locus 16q22 with overall IS in this study is driven by a subset of patients with another IS subtype that may also have as yet undiscovered atrial fibrillation or cardioembolic stroke. In addition, significant associations of genetic risk scores for atrial fibrillation with overall IS were recently found to be almost entirely explained by an association with cardioembolic stroke^19^.

Several factors may have prevented us from being able to replicate all associations investigated here. First, we had limited power to discover (nominal) associations in a relatively limited cohort size; our power was particularly limited for lower-frequency variants or variants with modest effect, a characteristic true of the vast majority of loci discovered through genome-wide association studies (stroke loci included). Thus, it is entirely possible that non-replicated loci are truly associated with overall IS and its subtypes and would replicate in larger sample collections. Second, failure to replicate may also be due to phenotypic heterogeneity; subtyping approaches vary across studies, and subtyping is imperfect, as many samples are categorized as ‘undetermined,’ thus allowing for potentially incorrectly subtyped cases (and consequently, reducing power). However, to decrease diagnostic uncertainty we excluded patients with transient ischemic attacks. Despite these limitations, most variants showed comparable effect sizes in the same direction as reported previously.

In conclusion, we validated two loci (4q25, 16q22) associated with IS caused by CE. In addition, our study may suggest that locus 16q22 may also be associated with overall IS or another subtype for which the current study may lack power to demonstrate a significant association. Future studies should search for the causal variants underlying these loci by fine-mapping and further discerning which genes within the loci may have functional consequence for disease.

## Electronic supplementary material

Supplemental data.
