# Author Correction: A replication study of genetic risk loci for ischemic stroke in a Dutch population: a case-control study

**DOI:** 10.1038/s41598-018-22952-z

**Published:** 2018-04-11

**Authors:** Allard J. Hauer, Sara L. Pulit, Leonard H. van den Berg, Paul I. W. de Bakker, Jan H. Veldink, Ynte M. Ruigrok, Dutch Parelsnoer Institute-Cerebrovascular accident (CVA) Study Group, Catharina J. M. Klijn, Ale Algra, Ewoud J. van Dijk, Peter J. Koudstaal, Gert-Jan Luijckx, Paul J. Nederkoorn, Robert J. van Oostenbrugge, Marieke C. Visser, Marieke J. Wermer, L. Jaap Kappelle

**Affiliations:** 1 Department of Neurology and Neurosurgery, Brain Center Rudolf Magnus, University Medical Center Utrecht, Utrecht, The Netherlands; 2 Department of Genetics, Center for Molecular Medicine, University Medical Center Utrecht, Utrecht, The Netherlands; 3 Department of Epidemiology, Julius Center for Health Sciences and Primary Care, University Medical Center Utrecht, Utrecht, The Netherlands; 4 The Dutch Parelsnoer Institute-Cerebrovascular accident (CVA) Study Group, Utrecht, The Netherlands; 5 Department of Neurology, Donders Institute of Brain Behaviour & Cognition, Center for Neuroscience, Radboud University Medical Center, Nijmegen, The Netherlands; 6 Department of Clinical Epidemiology, Leiden University Medical Center, Leiden, The Netherlands; 7 Department of Neurology, Erasmus Medical Center, Rotterdam, The Netherlands; 8 Department of Neurology, University Medical Center Groningen, Groningen, The Netherlands; 9 Department of Neurology, Academic Medical Center, Amsterdam, The Netherlands; 10 Department of Neurology, Maastricht University Medical Center, Maastricht, The Netherlands; 11 Department of Neurology, VU Medical Center, Amsterdam, The Netherlands; 12 Department of Neurology, Leiden University Medical Center, Leiden, The Netherlands

Correction to: *Scientific Reports* 10.1038/s41598-017-07404-4, published online 22 September 2017

This Article contains typographical errors in the Acknowledgements section.

“Ruigrok was supported by a clinical fellowship grant of the Netherlands Organization for Scientific Research (NWO) (project no. 40-00703-98-13533).”

should read:

“Ruigrok was supported by a clinical fellowship grant of the Netherlands Organization for Scientific Research (NWO) (project no. 90714533).”

