# Supplementary material for: A replication study of genetic risk loci for ischemic stroke in a Dutch population: a case-control study
Source: Sci Rep. 2017 Sep 22;7:12175. doi: 10.1038/s41598-017-07404-4 (PMC5610184; doi:10.1038/s41598-017-07404-4)
Supplement: Supplementary file 1 — Supplemental data. [file 41598_2017_7404_MOESM1_ESM.pdf]

Supplemental Information

A replication study of genetic risk loci for ischemic stroke in a Dutch population: a case-control study

Allard J. Hauer[1], MD; Sara L. Pulit[1,2], PhD; Catharina J.M. Klijn[1,3], MD, PhD; Ale Algra[1,4,5], MD, PhD; Leonard H van den Berg[1], MD, PhD; Ewoud J. van Dijk[3], MD, PhD; Peter J. Koudstaal[6], MD, PhD; Gert-Jan Luijckx[7], MD, PhD; Paul J. Nederkoorn[8], MD, PhD; Robert J. van Oostenbrugge[9], MD, PhD; Marieke C. Visser[10], MD, PhD; Marieke J. Wermer[11], MD, PhD; L. Jaap Kappelle[1], MD, PhD; Paul I.W. de Bakker[2,4], PhD; Jan H. Veldink[1], MD, PhD; Ynte M. Ruigrok[1*], MD, PhD; On behalf of the Dutch Parelsnoer Institute-Cerebrovascular accident (CVA) Study Group.

Supplementary Table S1. Baseline characteristics of the patients with ischemic stroke

	**n=1368**
Male	803 (58.7)
Age, median (IQR)	67.3 (56.5-77.2)
Cardiovascular history:	
- Previous stroke	331/1307 (25.3)
Cardiovascular risk factors:	
- Hypertension	750/1351 (55.5)
- Hyperlipidemia	432/1336 (32.3)
- Diabetes mellitus	209/1355 (15.4)
- Smoking	392/1287 (30.5)
- Family history	336/987 (34.0)
- Body mass index, mean (SD)*	26.3 (4.4)
TOAST classification:	
- Large artery atherosclerosis	362/1347 (26.9)
- Small vessel occlusion	287/1347 (21.3)
- Cardioembolic stroke	221/1347 (16.4)
- Other determined stroke	101/1347 (7.5)
- Undetermined stroke	376/1347 (27.9)

Data are in number (%) unless otherwise specified. IQR, interquartile range; SD, standard deviation.

* Percentage of missing values was 26.9.

Supplementary Table S2. Results of replication of previously established SNPs with ischemic stroke and its subtypes excluding individuals with missing genotypes

Phenotype	SNP	Nearest gene	Chr.	Base pair position	Previously reported association	Ref.	Cases/ Controls	Risk allele	RAF GoNL	RAF cases/ controls	Power (%)	Adj. OR (95% CI)*	p-value†
Overall IS	rs2200733	*PITX2*	4q25	110789013	1.25 (1.15-1.37), p = 3.14 x10^{-7}	2	1292/1416	T	0.10	0.11/0.10	11	1.05 (0.89-1.25)	1.00
Overall IS	rs505922	*ABO*	9q34	136149229	1.07 (1.03-1.11), p = 0.0006	8	1292/1416	C	0.34	0.33/0.32	37	1.08 (0.96-1.21)	0.85
Overall IS	rs10744777	*ALDH2*	12q24	111795214	1.10 (1.07-1.13) p = 7.12 × 10^{-11}	3	1292/1416	T	0.65	0.67/0.66	7	1.02 (0.91-1.14)	1.00
Overall IS	rs7193343	*ZFHX3*	16q22	72995261	1.11 (1.04-1.17), p = 0.00054	9	1292/1416	T	0.17	0.20/0.17	94	1.21 (1.05-1.39)	0.070
LAA	rs12122341	*TSPAN2*	1p13.2	115113069	1.19 (1.12-1.26), p = 1.30 × 10^{-9}	1	346/1416	G	0.26	0.26/0.24	34	1.13 (0.93-1.37)	0.90
LAA	rs556621	*SUPT3H/ CDC5L*	6p21.1	44626422	1.62 (1.36-1.93), p = 3.92 × 10^{-8}	4	346/1416	T	0.30	0.28/0.32	38	0.88 (0.73-1.06)	0.84
LAA	rs11984041	*HDAC9*	7p21.1	18992312	1.42 (1.28-1.57), p = 1.87 × 10^{-11}	5	346/1416	T	0.09	0.13/0.10	84	1.35 (1.04-1.74)	0.20
LAA	rs2107595	*HDAC9*	7p21.1	19009765	1.39 (1.27-1.53), p = 2.03 × 10^{-16}	6	346/1416	A	0.14	0.20/0.17	63	1.22 (0.98-1.51)	0.50
LAA	rs2383207	*CDKN2B-AS1*	9p21.3	22115960	1.16 (1.04-1.29), p = 0.0083	7	346/1416	G	0.50	0.53/0.48	79	1.23 (1.03-1.46)	0.17
LAA	rs505922	*ABO*	9q34	136149229	1.23 (1.07-1.18), p = 0.001	8	346/1416	C	0.34	0.35/0.32	47	1.15 (0.97-1.37)	0.74
SVD	rs10744777	*ALDH2*	12q24	111795214	1.17 (1.11-1.23), p = 2.92 × 10^{-9}	1	270/1416	T	0.65	0.69/0.66	33	1.13 (0.93-1.37)	0.98
CE	rs2634074	*PITX2*	4q25	110755885	1.33 (1.24-1.42),	3	212/1416	T	0.18	0.26/0.19	96	1.47	0.0159

					p = 1.52 $\times 10^{-16}$							(1.16-1.88)	
CE	rs2200733	*PITX2*	4q25	110789013	1.54 (1.33-1.78), p = 8.05 $\times 10^{-9}$	2	212/1416	T	0.10	0.14/0.11	62	1.32 (0.97-1.80)	0.51
CE	rs505922	*ABO*	9q34	136149229	1.13 (1.11-1.15), p = <0.001	8	212/1416	C	0.34	0.34/0.32	46	1.19 (0.95-1.48)	0.73
CE	rs7193343	*ZFHX3*	16q22	72995261	1.22 (1.10-1.35), p = 0.00021	9	212/1416	T	0.17	0.24/0.17	99	1.60 (1.24-2.06)	0.0021

SNP; single nucleotide polymorphism; Chr, chromosome; LAA, large artery atherosclerosis; IS, ischemic stroke; CE, Cardioembolism; SVD, small vessel disease; Ref, reference; RAF, risk allele frequency; GoNL, Genome of the Netherlands; Adj, adjusted; OR, odds ratio; CI, confidence interval.

* Adjusted for sex and age.

† Calculated by permutation.

REFERENCES

1. NINDS Stroke Genetics Network (SiGN), I. S. G. C. (ISGC). Loci associated with ischaemic stroke and its subtypes (SiGN): a genome-wide association study. *Lancet. Neurol.* **15,** 4–7 (2015).

2. Gretarsdottir, S. *et al.* Risk variants for atrial fibrillation on chromosome 4q25 associate with ischemic stroke. *Ann. Neurol.* **64,** 402–409 (2008).

3. Kilarski, L. L. *et al.* Meta-analysis in more than 17,900 cases of ischemic stroke reveals a novel association at 12q24.12. *Neurology* **83,** 678–85 (2014).

4. Holliday, E. G. *et al.* Common variants at 6p21.1 are associated with large artery atherosclerotic stroke. *Nat. Genet.* **44,** 1147–51 (2012).

5. Traylor, M. *et al.* Genetic risk factors for ischaemic stroke and its subtypes (the METASTROKE Collaboration): A meta-analysis of genome-wide association studies. *Lancet Neurol.* **11,** 951–962 (2012).

6. Bellenguez, C. *et al.* Genome-wide association study identifies a variant in HDAC9 associated with large vessel ischemic stroke. *Nat. Genet.* **44,** 328–33 (2012).

7. Gschwendtner, A. *et al.* Sequence variants on chromosome 9p21.3 confer risk for atherosclerotic stroke. *Ann. Neurol.* **65,** 531–539 (2009).

8. Williams, F. M. K. *et al.* Ischemic stroke is associated with the ABO locus: the EuroCLOT study. *Ann. Neurol.* **73,** 16–31 (2013).

9. Gudbjartsson, D. F. *et al.* A sequence variant in ZFHX3 on 16q22 associates with atrial fibrillation and ischemic stroke. *Nat. Genet.* **41,** 876–8 (2009).
